# Nitric-Acid Oxidized Single-Walled Carbon Nanohorns as a Potential Material for Bio-Applications—Toxicity and Hemocompatibility Studies

**DOI:** 10.3390/ma14061419

**Published:** 2021-03-15

**Authors:** Wojciech Zieba, Joanna Czarnecka, Tomasz Rusak, Monika Zieba, Artur P. Terzyk

**Affiliations:** 1 Physicochemistry of Carbon Materials Research Group, Faculty of Chemistry, Nicolaus Copernicus University in Torun, Gagarin Street 7, 87-100 Torun, Poland; wuzet95@gmail.com (W.Z.); m.zieba995@gmail.com (M.Z.); 2 Department of Biochemistry, Faculty of Biological and Veterinary Sciences, Nicolaus Copernicus University in Torun, Lwowska Street 1, 87-100 Torun, Poland; j_czar@umk.pl; 3 Department of Physical Chemistry, Faculty of Pharmacy, Medical University of Bialystok, Kilinskiego Street 1, 15-328 Bialystok, Poland; tomasz.rusak@umb.edu.pl

**Keywords:** carbon nanohorns, oxidation, toxicity, hemocompatibility, biocompatibility

## Abstract

The results of in vitro studies of single-walled carbon nanohorn (SWCNH) oxidized materials’ cytotoxicity obtained by the cell membrane integrity (Neutral Red Uptake (NRU)) and metabolic activity (by 3-(4,5-dimethylthiazol-2-yl)-2,5-diphenyltetrazolium bromide (MTT)) on A549 and human dermal fibroblasts (HDF) cell lines are presented. We also present hemocompatibility studies on human and porcine blood, and an erythrocyte concentrate to prove that the obtained samples will not interfere with blood components. Characterization of the materials is supplemented by ζ-potential measurements, Transmission Electron Microscope (TEM) imaging, and thermogravimetric studies (TG). The presented results show the correlation between the specific surface area of materials and the platelet aggregation, when the I_D_/I_G_ ratio determined from Raman spectra correlates with hemoglobin release from the erythrocytes (in whole blood testing). A plausible mechanism explaining the observed correlations is given. The cytotoxicity and hemocompatibility studies prove that the studied materials are acceptable for use in biomedical applications, especially a sample SWCNH-ox-1.5 with the best application potential.

## 1. Introduction

Single-walled carbon nanohorns (SWCNHs) are aggregated spherical nanoobjects with an outer diameter of 40–50 nm, created by conical-shaped structures, where a single nanohorn is a closed cone with the diameter 2–5 nm [1]. After oxidation, so-called “nano-windows” [2,3] (or “nanogates”) are created, i.e., nanometric holes opening the internal space of a single nanohorn and inducting significant increase in the specific surface area value as well as improving adsorption capacity.

According to the results of our previous study [4], nitric acid-treated SWCNHs used as electrodes have great accessibility to surface area for electrolyte ions. In combination with the great stability of the obtained electrodes, huge specific surface areas, and pore distribution between 2–5 nm, SWCNHs seem to have great potential toward sensing and, especially, biosensing.

Carbon nanomaterials exhibit four main properties important in electrochemical biosensors: electrochemical activity, electrical conductivity, large surface area, and the ability of easy functionalization [5]. The previously presented materials [4] are proven to fulfil those conditions, but there are two additional that should be fulfilled, namely biocompatibility [5] as well as hemocompatibility. This is because, generally, each material used in biomedical applications and subjected to contact with a human body should be nontoxic and should have negligible impact on the functioning of the organism.

There are already reports in the literature about the toxicity of pristine SWCNHs during tests both in vitro [6,7,8], on different cell lines, and in vivo during respiratory, oral, and skin or eye sensitization [9,10]. It is also proven that SWCNHs are getting inside cells [7,11,12,13], but no cancerogenic/mutual activity was observed [9]. However, after modification, the toxicity can change and it is hard to predict the direction of change. Even oxidation of the material can be performed in many different ways (for example, with oxygen [14,15,16,17,18], hydrogen peroxide [19,20,21,22], or plasma [23] and other [24,25,26,27,28,29,30,31,32,33] treatments), resulting in changes in different properties by incorporating various heteroatoms; therefore, it is important to prove that toxicity of the corresponding material is still low. Especially considering the reports about the biosensing applications of SWCNH, there is a lack of investigations on the safety of the used materials [20,34,35,36,37,38,39,40,41]. Similar to cytotoxicity, hemocompatibility [42,43,44] studies are also important, especially considering materials dedicated to biosensor applications. The ability to perform measurements in terms of whole blood is crucial especially for glucose meters and for many others purposes [45]. Therefore, it is necessary to investigate eventual blood cell coagulation, which could effectively prevent a sensor from working or can cause erythrocyte damage, which are dangerous for the whole organism.

To prove that the obtained materials are acceptable for use in biomedical applications, especially as biosensors, the results of in vitro studies of cytotoxicity by the cell membrane integrity (Neutral Red Uptake (NRU)) and metabolic activity (3-(4,5-dimethylthiazol-2-yl)-2,5-diphenyltetrazolium bromide (MTT)) tests on A549 and human dermal fibroblasts (HDF) cell lines are presented in this study. Additionally, hemocompatibility studies were performed to prove that the obtained samples will not interfere with blood components. Characterization of the materials is supplemented by additional ζ-potential measurement and thermogravimetric studies. In addition, an attempt to correlate the biological effects with specific material properties was performed, leading to a plausible mechanism explaining the observed results.

## 2. Materials and Methods 

### 2.1. Synthesis and Characterization of Materials

**Figure 1:**
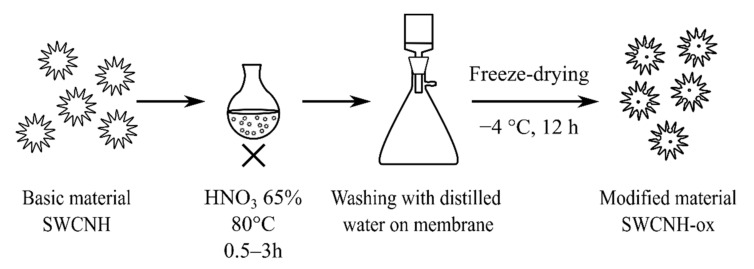
Schematic representation of the studied sample preparation.

SWCNHs (NEC, Minato, Japan) were oxidized as it was previously reported [4] by 65% nitric acid at 80 °C for different times marked as SWCNH-ox-y (where y = 0.5, 1, 1.5, and 3 h), were washed, and were freeze-dried (Figure 1). The samples were characterized by thermogravimetry (in N_2_, heating rate 10 °C/min, three repetitions, Netzsch, Jupiter STA 449 F5) and ζ potential (25 °C, Particulate Systems, NanoPlus HD, Micromeritics, Norcross, GA, USA) at different pH levels (0.01 mg/mL solution in deionized water with an ionic strength 0.1 M using HCl, NaOH, and nKCl—all analytical grade, Chempur, Piekary Śląskie, Poland) as an extension of previously presented results [4]. The ζ-potential studies for the initial SWCNHs were impossible to perform due to the lack of dispersion in water. Additionally, the transmission electron microscopy (TEM) (FEI, Tecnai F20X-Twin, Particulate Systems, Norcross, GA, USA) images of all samples were prepared to obtain deeper insight into the observed changes caused by oxidation.

### 2.2. In Vitro Studies

#### 2.2.1. Viability Assays

The cell viability of human dermal fibroblasts (HDF) and human A549 cell lines were determined based on their metabolic activity using the MTT assay. The cells were cultured in Dulbecco’s Modified Eagle Medium (DMEM) or Ham’s F12 medium, respectively, with 10% Fetal Bovine Serum (FBS) (Biowest) under sterile conditions at 37 °C and 4.9% CO_2_. The cells were seeded on 48-well plates at a density of approximately 3 × 104 cells/well in 0.3 mL medium for 24 h. Then, the culture medium was replaced with a medium containing SWCNH-ox materials (1–1000 µg/mL), and the cells were incubated for an additional 24 h. To be sure that the materials were well-dispersed, the solutions were sonificated and mixed using votex. With that procedure, even SWCNHs, which is highly hydrophobic, were well-dispersed. Subsequently, the cells were washed with Phosphate-Buffered Saline (PBS) and incubated with 0.5 mg/mL MTT (Sigma-Aldrich, Darmstadt, Germany) at 37 °C for 30 min. The Obtained formazan crystals were dissolved in 0.5 mL of Dimethyl sulfoxide (DMSO). Absorbance was measured using a microplate reader (Epoch3, BioTek Instruments, Winooski, VT, USA) at 570 nm. The reduction of cell viability was calculated as a percentage normalized to the control cells’ viability. Each experiment was repeated at least 3 times.

#### 2.2.2. NRU

After incubation with SWCNH-ox materials, the cells were washed with PBS and 0.033% neutral red in fresh medium was added to each well. After 45 min of incubation at 37 °C, the dye was removed and the cells were washed with warm PBS. The uptaken dye was released from the cells by adding 1% acetic acid in 50% ethanol and analyzed spectrophotometrically (Epoch3, BioTek Instruments, Winooski, VT, USA) at 540 nm. The number of living cells was normalized as a percentage of control cells. Each experiment was repeated at least 3 times.

### 2.3. Hemocompatibility Studies

#### 2.3.1. Platelet Aggregation In Vitro

Platelets aggregation was evaluated following the impedance method, as previously described using a Whole Blood Lumi-Aggregometer (Chronolog, Havertown, PA, USA) [36]. The whole human blood (500 µL) was diluted 1:1 in saline and thermostated at 37 °C in aggregometer. The final concentration of platelets was found to be ≈1 × 10^11^ L^−1^. Blood samples were incubated with SWCNH-ox materials (10–250 µg/mL) or saline, and impedance changes were measured for 10 min. Then, collagen-stimulated (Chrono-Par Collagen, 2 µg/mL) platelet aggregation was evaluated.

#### 2.3.2. Hemolysis Investigation

The hemolysis investigation studies were performed using human blood, porcine blood and washed erythrocytes suspension (Hematocrit (HCT) ~15%) obtained from porcine blood. The samples were incubated with SWCNH-ox materials (1–250 µg/mL) for 30 min at 37 °C, then centrifuged at 200× *g* for 15 min. Supernatants were again centrifuged at 13,600× *g* for 6 min. The hemoglobin concentration in the received plasma was measured spectrophotometrically (Helios Gamma, Spectronic Unicam, Leeds, UK) according to the methods of Fairbanks VF, Ziesmer SC, O’ Brien PC for measuring plasma hemoglobin in micromolar concentration [46].

Hemolysis was measured by determining the free hemoglobin (fHb) released into the surrounding media, including human and porcine blood, and washed erythrocytes suspension (HCT ≈15%) obtained from porcine blood. The samples were incubated with SWCNH-ox materials (1–250 µg/mL) for 30 min at 37 °C and then centrifuged at 200× *g* for 15 min. The top (upper) of the supernatant layer was carefully removed using a plastic Pasteur pipette and was further centrifuged at 12,000× *g* for 5 min. The level of hemoglobin was measured spectrophotometrically (Helios Gamma, Spectronic Unicam, Leeds, UK) using the 3-wavelength method described by Fairbanks et al. [46].

## 3. Results

### 3.1. Physicochemical Characterisation of Materials

**Figure 2:**
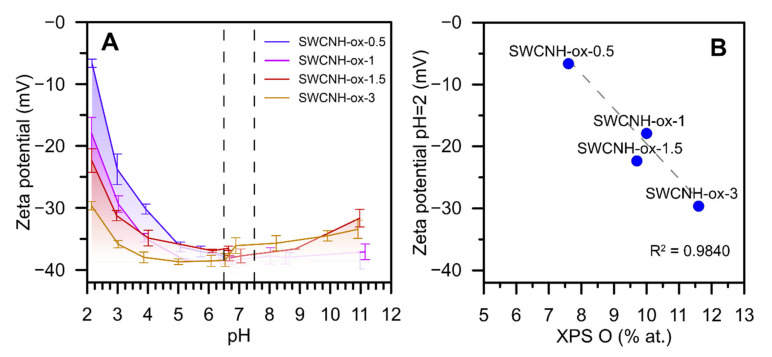
The results of ζ-potential analysis as a pH function at 25 °C (**A**). Figure (**B**) shows the correlation between ζ-potential at pH = 2 and XPS-determined oxygen content. Determination coefficient was calculated for three samples (single-walled carbon nanohorn (SWCNH)-ox-0.5; 1; 3).

**Figure 3:**
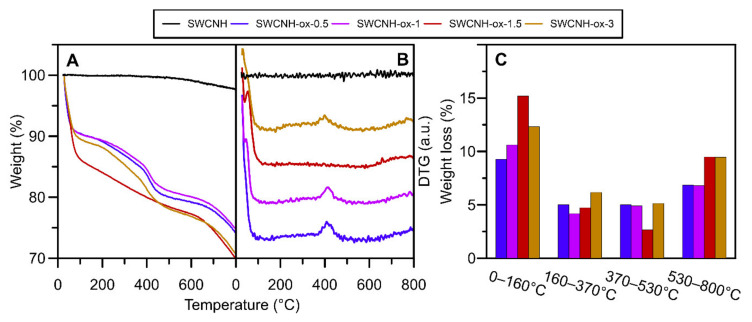
The results of thermogravimetric studies of SWCNH and SWCNH-ox material heating in N_2_ (**A**). Panel (**B**) shows the derivative (DTG) curves. Panel (**C**) shows the weight loss in each characteristic temperature range for materials.

**Figure 4:**
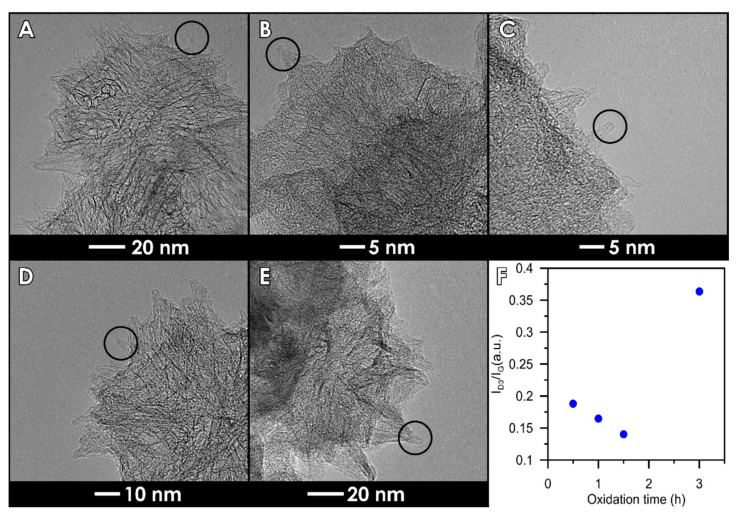
Transmission Electron Microscope (TEM) images of the materials: (**A**) SWCNH, (**B**) SWCNH-ox-0.5, (**C**) SWCNH-ox-1, (**D**) SWCNH-ox-1.5, and (**E**) SWCNH-ox-3. Circles show the tip shapes and an amorphous carbon evaluation through the oxidation process. (**F**) Dependence of I_D3_/I_G_ on the oxidation time.

Some basic characteristics of SWCNH-ox (e.g., porosity, chemical composition of surface groups by X-ray Photoelectron Spectroscopy (XPS) and Fourier Transform Infrared Spectroscopy (FTIR), Raman spectra, etc.) were reported previously [4]; however, the results of the ζ-potential analysis in broad pH range (Figure 2) as well as the results of thermogravimetric (Figure 3) analysis and more detailed TEM studies (Figure 4) are new and have not been published yet. Additionally, we add the atomic concentration from XPS measurements (see Table 1) and I_D3_/I_G_ ratio calculated from previously published Raman spectra [4].

The measurements of ζ-potential in a wide pH range give the information not only about the changes in an electrostatic potential of the samples but also about its chemical character. From Figure 1, one can conclude that the largest differences between materials are observed at the acidic pH. In the physiological pH region (marked on a chart), there are no significant differences; however, this analysis proves that obtained materials are gradually oxidized. Moreover, at the low pH region, the values of ζ-potential become more negative with the rise in oxidation time (Figure 2A). It is visible that the sample SWCNH-ox-1.5 differs from overall correlations presented on Figure 2B. It may be caused by the different nature of oxygen groups leading to its higher acidity.

The thermogravimetric studies of carbon nanomaterials were performed to investigate thermal stability of the samples, but the profiles of the obtained curves also showed some information about the composition of the materials. As one can observe on the surface of initial SWCNH, the amount of oxygen functional groups is negligibly small and this is why the material is strongly hydrophobic, showing almost any changes on the thermogravimetric data determined in N_2_ atmosphere through all temperature range (Figure 3). The first decrease observed between 0–160 °C is responsible for the release of water captured by studied materials. The second peak (in the range of 160–370 °C) proves the presence of weekly acidic functional groups, mostly carboxyls [37,47] on the surface. The next range (370–530 °C) corresponds to groups such as lactones, anhydrides, as well as an amorphous carbon [38] covering the structures after oxidation. The last range between 530 and 800 °C represents desorption of the most temperature-resistant groups: anhydrides, lactones, phenols, carbonyls, quinones, and ethers [37,47]. It is important to note that lactones and anhydrides have different decomposition temperature ranges dependent of their location and chemical surrounding the surface of the sample. In specific conditions, they can rearrange to groups with higher thermal stability. 

For one of the studied samples, namely SWCNH-ox-1.5, weight loss in the range of 370–530 °C clearly decreases. We suggest that it can be the result of two effects that occur: first, decreased content of amorphous carbon on sample, which is confirmed by TEM and Raman spectra. The agglomerates, as well as single cones look different after subjected to different oxidation times visible in the images (Figure 4). The individual nanohorns are additionally marked by circles in Figure 4 to draw attention to the changes in the structure (opened tips lead to increased specific surface area of the materials [4]) and the clearness of the tip visibility in each sample due to the amorphous carbon presence. Additional studies of Raman spectra presented in previous work [4] show differences in amorphous carbon content. The spectra were deconvoluted according to the Sadezky method [48]. The presence of the D3 band (≈1500 cm^−1^) is connected to amorphous carbon and it increases with its content. The I_D3_/I_G_ ratios were calculated based on the deconvoluted peak area, and the results are presented in Figure 4F. It shows agreement with the TEM images, where we can observe the decreasing content of amorphous carbon up to the sample SWCNH-ox-1.5 and an increase for the last one. The second effect is connected to the presence of oxygen-containing groups on the surface of carbon nanohorns. The thermal stability of groups is highly dependent on its location, so the same group can be decomposed in different temperatures due to the transformations of groups. Additionally, it is important to note that low weight loss in the range 370–530 °C is compensated in the next range, where we observe an increase in weight desorbed (Figure 3C). We suggest that, during heating, oxygen functional group transformations occur, leading to the formation of higher thermal stability groups [47].

Summing up, the results collected in Figure 2, Figure 3 and Figure 4 confirm the presence of surface oxygen—containing functionalities as well as leading to the conclusion about increasing structure disorder of the SWCNH aggregates caused by increasing oxidation time. This is a confirmation of the results of the Raman spectra measurements [4] showing progressive increase in the I_D_/I_G_ (intensity ratio between disorder and graphitization band on the Raman spectra) ratio with increasing oxidation time. The results of the ζ-potential measurements confirm the differences between the concentration of surface oxygen groups for studied samples; however, they also demonstrate a similarity in the surface charge for all studied samples in the range of neutral pH (ζ = c.a. −37 mV).

### 3.2. In Vitro Toxicity Studies Results

The toxicity of obtained materials was tested via in vitro studies to prove its biocompatibility and investigates eventual harmful interactions with cells in a very basic way. Unmodified SWCNH decreased the cell viability shown by NRU in both of the tested cell lines. However, the mitochondrial activity tested with MTT was affected only in the A549 line in a concentration-dependent manner.

**Figure 5:**
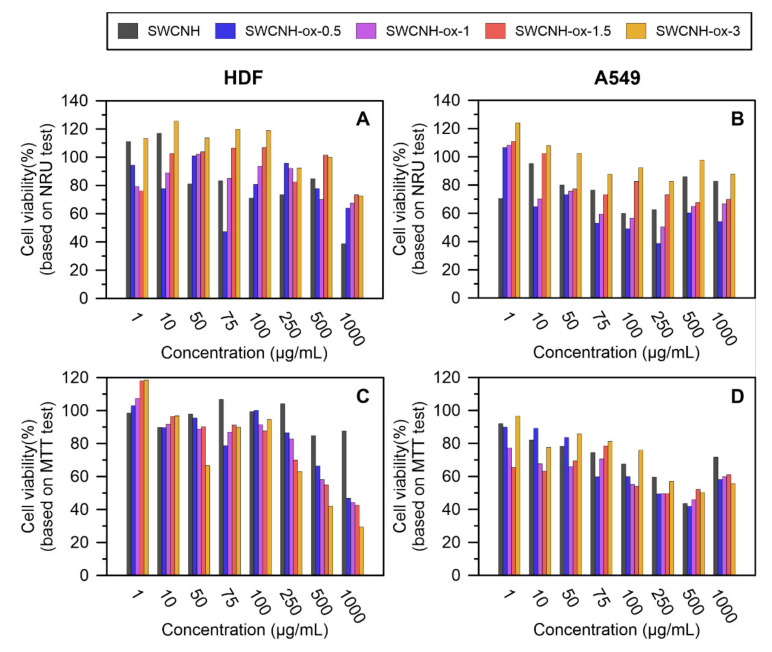
The results of in vitro experiments (**A**) Neutral Red Uptake (NRU) on human dermal fibroblasts (HDF), (**B**) NRU on A549, (**C**) 3-(4,5-dimethylthiazol-2-yl)-2,5-diphenyltetrazolium bromide (MTT) on HDF, and (**D**) MTT on A549 with SWCNH and SWCNH-ox materials.

Modification of the material by oxidation also influences cell growth and viability, and these effects depended on oxidation time. The highest toxicity—smallest number of viable cells, determined using the NRU test, and the lowest cell viability, confirmed by the MTT assay—was found using material oxidized for 0.5 h. In this case, 100 µg/mL and 50 µg/mL were below the half maximal effective concentration (EC50) for HDF and A549 cells, respectively. An extension of the oxidation time to 3 h increased the biocompatibility of the material to human dermal fibroblasts as well as epithelial A549, showing higher viability in the NRU test compared to analogous concentrations of the control material. Moreover, SWCNH-ox-3 at a concentration up to 100 µg/mL had properties similar to unmodified SWCNH and did not significantly affect the mitochondrial activity of the tested cell lines. Above this concentration, the effect on the MTT-based viability of HDF fibroblasts was significantly dependent on the oxidation time, and the toxic effect was confirmed at a concentration of 500 µg/mL for the material oxidized for 3 h (Figure 5A). Epithelial A549 cells responded with decreased viability, relative to HDF cells, at lower concentrations of test materials (Figure 5B). Regardless of the material oxidation state, the decrease in their metabolic potential was observed in the presence of 250 µg/mL of SWCNH.

### 3.3. Hemocompatibility Studies Results

The hemocompatibility investigation of studied materials was carried out to prove the general safety of using in contact with blood in many potential biomedical applications and to find out the safe range of the materials’ concentration for such applications.

**Figure 6:**
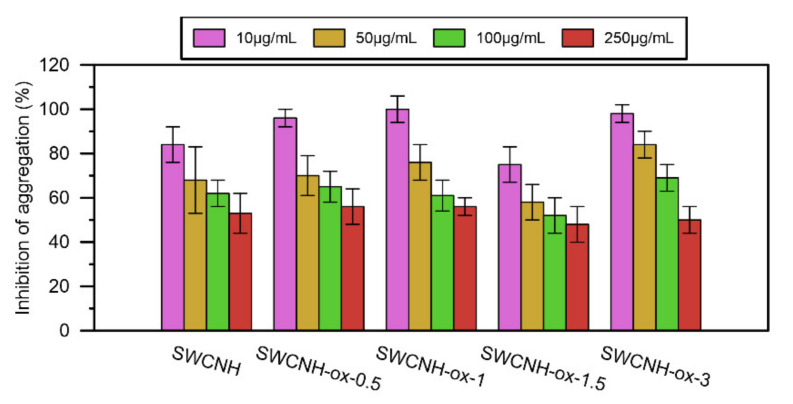
The results of the inhibition of platelet aggregation by SWCNH-ox materials in concentrations 10–250 µg/mL compared to the control (100%).

The results of the platelet aggregation for each nanomaterial in comparison to the control are presented on Figure 6. Before the addition of collagen to the samples, none of the tested materials showed an increase in impedance due to the aggregation process, and with collagen, all obtained impedance results are lower than for the control (at one point equal). At the same time, with increasing SWCNH-ox concentration, samples present significant decreases in platelet aggregation, up to 50% compared with the control.

**Figure 7:**
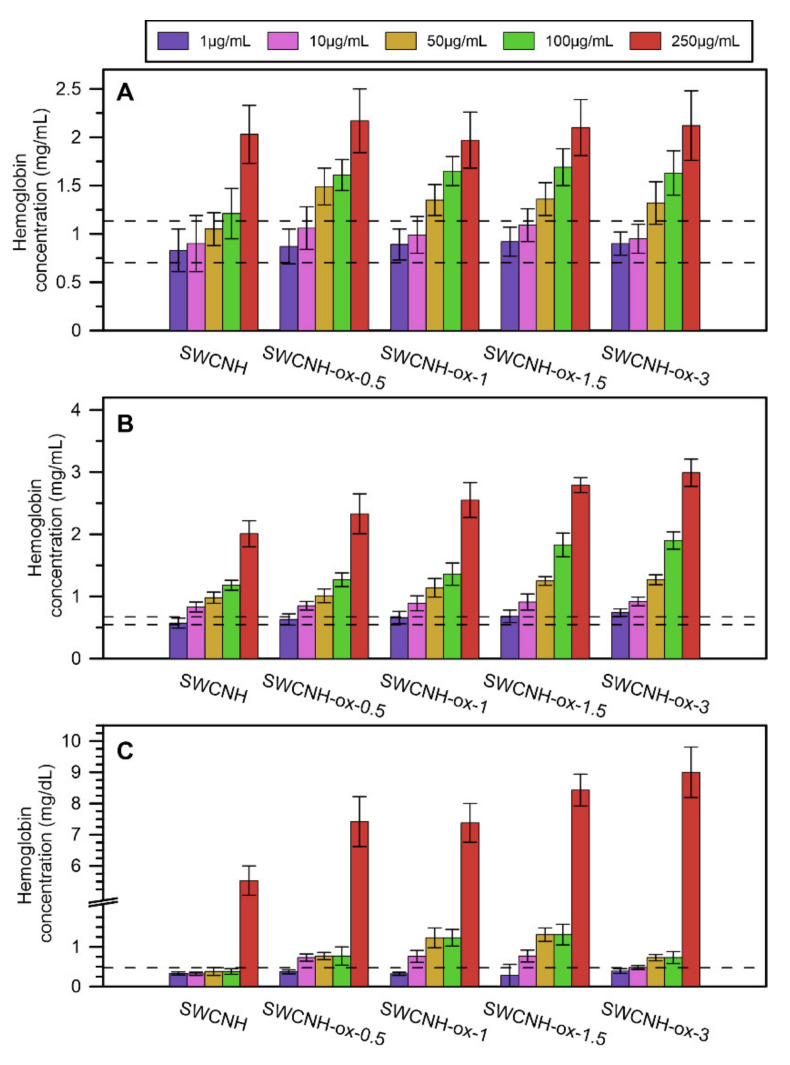
The results of hemolysis investigation by SWCNH-ox materials in different concentrations, 1–250 µg/mL, on (**A**) human blood, (**B**) porcine blood, and (**C**) erythrocyte suspension obtained from porcine blood. Dotted lines represent the range of data for the control.

The hemolysis investigation presented in Figure 7 for human blood (a), porcine blood (b), and erythrocyte suspension (c) shows that, in low concentrations of SWCNH-ox materials that do not cause apparent system malfunction, the results are in the range of the control results or slightly above. This proves that some erythrocytes can be slightly damaged, but only at the concentration of 250 µg/mL can all materials cause noticeable increase in the release of hemoglobin.

## 4. Discussion

The studied materials (SWCNH-ox) are remarkably different considering the amount of surface functional groups and their structure. The only change in the modification process was the time of oxidation; however, the ζ potential analysis (Figure 2A) (especially in the acidic region) shows the increasing amount of acidic groups on the surface of materials with increasing oxidation time. This is why we observe the correlation between ζ potential at pH = 2 and oxygen content (Figure 2B). As it was shown before [4] based on the FTIR and XPS data, remarkable differences in the concentration of surface hydroxylic groups are observed with the rise in oxidation time (i.e., the concentration of surface OH groups remarkably increases with the oxidation time). This can be the reason for the observed differences in surface charge at low pH limits. Moreover, since the acidity of a group depends on its location on a surface, we can see that a short oxidation time leads to the production of weaker acidic groups (after adsorption of protons at the acidic pH, the surface has a larger charge).

The complexity of the oxidation process is better shown on thermogravimetric curves (Figure 3), leading to the conclusion that SWCNH-ox materials can be divided into two groups due to the shape of the TG curves. The first group contains the samples possessing amorphous carbon residues on the surfaces (i.e., SWCNH-ox-0.5, SWCNH-ox-1, and SWCNH-ox-3); the second group (containing only one sample, i.e., SWCNH-ox-1.5) is the sample with mostly strong acidic group presence, and this structure is residues-free. It is clearly visible on TEM images presented on Figure 4. The circled regions point to the tips of the SWCNH (Figure 4B,C,E) where non-regular carbon impurities are visible, compared to its absence on Figure 4D. Also important is the core visibility in each sample. In the reference sample (Figure 4A), the cross section is clean and the internal structure of the agglomerate is clearly visible. A very similar situation occurs for the sample SWCNH-ox-1.5 (Figure 4D). However, for the samples SWCNH-ox-0.5, 1, and 3, the internal structure of the agglomerates is almost invisible. It is associated with the partial structure destruction during oxidation for samples SWCNH-ox-0.5 and -ox-1, leading to amorphous carbon creation and, further, additional burning-off of this residuals during longer oxidation (for 1.5 h).The longest oxidation time again leads to structure destruction (for the time = 3 h), which causes back appearance of amorphous carbon.

The cytotoxicity of the materials was investigated on two cell lines: A549 (adenocarcinomic human alveolar basal epithelial cells—model of airway epithelium) and HDF (dermal fibroblast—normal human cells). The observed toxicity of the tested materials is generally low, what is justified and proven in the literature for SWCNH-based materials [9,39]. It is also important to mention that, generally, spherical carbon nanoobjects are reported as less toxic than nanotubes or nanowires because of the limited (by the shape) adhesion to the cells [49]. This proves the great potential of SWCNH-based materials toward biomedical applications and the importance of further biocompatibility research to fully understand the occurring phenomenon.

SWCNH-ox at the lowest concentration, 1 µm/mL, affects the number and metabolism of cells, which is particularly visible in the HDF line (Figure 5). It is possible that, at this concentration, nonspecific bonds between the functional groups of the material and the receptors in the cell membrane are interpreted by the cells as mobilizing and/or stimulating signals. Up to 250 µg/mL, SWCNH-ox with smaller surfaces are more conducive to cell viability in the range close to the control. Smaller surfaces of materials do not significantly affect the interaction with cells and plasma proteins, which is often the suggested cause of the mitochondrial toxicity of carbon materials [40].

At high concentrations of nanohorns (>250 µg/mL), the oxidation associated with an increase in carbon surface disorder reduces the mitochondrial potential shown by the MTT test. This effect may be caused by penetration of the material inside the cell (a disorder/amorphous material will cross or disturb the membrane barrier more easily) and adsorption on/block proteins essential for the regulation of metabolism [50].

Safe concentrations for all oxidized materials are defined as lower than 250 µg/mL (based on the cytotoxicity results); thus, that range of concentrations was used for the hemocompatibility investigation. The results obtained for the studied samples indicate that the materials do not show the ability for platelet aggregation, even in the presence of the coagulant (collagen). On the contrary, they show the ability to reduce aggregation in a significant way compared to the control (Figure 6). The concentrations 50 µg/mL and 100 µg/mL slightly increase hemolysis but, at the same time, reduce the aggregation of platelets by about 30–40%. This effect is stronger at higher concentrations; however, generally, the best performance occurs for sample SWCNH-ox-1.5.

Including hemocompatibility studies, the safe range for using SWCNH-ox materials in human body is lowered to 100 µg/mL due to a relatively high hemolysis effect for the highest concentrations. Especially, the results obtained for erythrocyte suspension show this limitation. However, the hemolysis of blood evoked by 10% water addition was 9.62 ± 0.20 mg/mL, almost five times more than the results presented for blood hemolysis by materials (excepting the erythrocyte suspension). 

**Figure 8:**
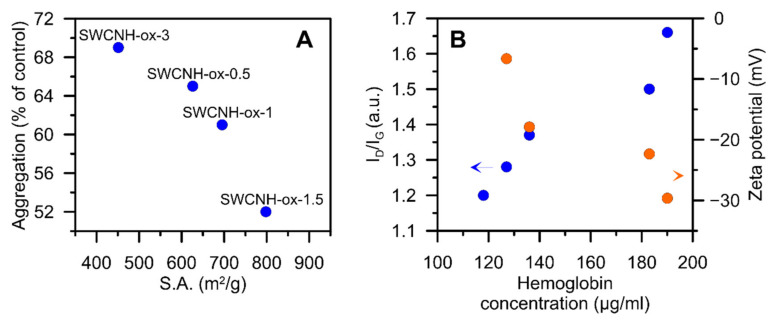
Correlation plots showing the dependence between (**A**) the aggregation of platelets and internal specific surface area [4] of SWCNH-ox materials and (**B**) the hemoglobin concentration from the hemolysis of porcine blood experiment and I_D_/I_G_ ratio from Raman spectra [4] of SWCNH-ox materials. Data for the materials’ concentration at 100 µg/mL.

As presented in Figure 8, the aggregation of platelets as well as hemolysis strongly depend on the physicochemical characteristic of the materials. The growing internal specific surface area [4] (i.e., the Brunauer–Emmett–Teller theory (BET) surface area diminished by the BET area determined for initial SWCNH) of the materials limits the platelet aggregation (Figure 8A). This can be associated with the increasing adsorption capacity of the materials. The SWCNH-ox in the blood may adsorb the aggregating factor on their surface (e.g., collagen) and consequently reduce the platelet aggregation.

Platelet aggregation is a process depending on platelet interaction with each other. However, during our measurements, it was observed that platelets also interact with the material. The increase in internal surface area is related proportionally to the increasing surface area originated from the edge carbon surface atoms located at nanowindow entrances. Thus, the internal surface area plotted on Figure 8A originates not only from the opening of SWCNH-ox internal channels but also from the appearance of nanowindow edge carbon atoms. It was well-documented by Kaneko et al. [51] that the edge carbon atoms can remarkably contribute to the surface area of carbon nanomaterials (see also [52]). Nanowindow edge carbon atoms play also important role in the rise of SWCNH specific capacitance, as it was proved recently [4]. Formed during oxidation, nanowindows, with oxygen containing functional groups located at the edges, are able to interact not only with free chemical compounds but also with receptor proteins and functional groups modifying the surface proteins of the cell membrane. In our opinion, platelet receptors and pro-aggregating factors can be physically immobilized/entrapped at the periphery of the nanowindows that are formed, using the exposed functional groups. Thus, we can state that we observe nanowindows—induced changes in platelet aggregation—and this is documented by the correlation present in Figure 8A. This effect is new and has not been reported yet. 

The second observed correlation occurs between the I_D_/I_G_ ratio determined from the Raman spectra [4] and hemoglobin release from the erythrocytes (in whole blood testing) (Figure 8B). In this case, one can see that the progressive destruction (damage) of the graphitic structure (see above) as well as a degree of functionalization cause more damage to the erythrocytes and increase the hemoglobin release. The erythrocytes’ membrane comprises an external layer, rich in carbohydrates, and a lipid bilayer, with numerous protein transporters, responsible for gas exchange and maintaining other cell functions [53,54]. On the cytoplasmic side of the lipid bilayer, an inner network of proteins forms the membrane skeleton [53]. The proteins of the membrane skeleton provide for the extremely high durability, flexibility, and deformability of the erythrocytes [53]. The normal zeta potential of the red blood cell is −15.7 mV [55]. Since the ζ potential of the tested materials at physiological pH ≈7 is similar for all tested materials, the observed correlation between oxidation and hemolysis at high concentrations of the tested materials is not related to the ionic interactions. However, progressive destruction of the carbon surface increases the possibilities for physical interaction with erythrocyte membrane proteins, and the resulting interactions can lead to mechanical damage to the erythrocytes’ membrane.

So far, in the literature, there are no results showing similar dependencies, so further investigations are needed to fully prove this thesis.

Over the years, carbon nanomaterials (CNM) have been employed in many different biomedical applications, mostly in biosensing, bio-imaging, drug and gene delivery systems, therapeutic materials, or tissue engineering [56]. However, each of the recently investigated CNMs has some unique properties, advantages, and disadvantages, making it more dedicated for exact application. The oxidized SWCNHs presented in this paper seem to collect most of the benefits needed for biosensing applications. So far, in this field, the most popular are carbon nanotubes (CNT) due to their high surface area and easy modification toward biomolecules binding [57,58]. The studied materials have similar or even greater specific surface area [4] but also are highly homogeneous (CNT shows differentiation not only in terms of morphology—diameters and length—but also the chemical character—metallic and semiconducting CNT). After the oxidation process, the presented materials obtain great stability in water suspension, similar to CD, CQD, or GQD, as well as a great amount of edge carbon atoms (due to nanowindow presence) and oxygenated groups [4,59] as it is in carbon black [57]. These changes allow the obtained materials to attach biomolecules and to induce better electrochemical sensing [57]. Graphene and its derivatives work in a similar way, also known as the least toxic carbon nanomaterials. Generally, the cytotoxicity of CNM is still not fully understood [56]. However, successive research may allow in the future to create a general toxicological statement depending on the properties of carbon nanomaterials.

As presented, SWCNH and especially its oxidized forms have many advantages and, in many ways, seem to be the best for biomedical applications. Additionally, SWCNHs have their own advantages for biosensing already reported in the literature, such as lower detection limits, better sensitivity, and selectivity compared with other CNM [57].

In this publication, for the first time, the hemocompatibility studies of pristine and oxidized SWCNHs are presented. Additionally, for the first time, the comprehensive research of SWCNH modified with nitric acid was carried out for biomedical applications. The presented results are promising; however, there is plenty of work to fully understand the processes behind the differences in toxicity and hemocompatibility of the shown materials. In future work, the TPD (Temperature Programmed Desorption) spectra will be also reported to assign the peaks from the thermogravimetric curves to surface groups more carefully, and the results will be reported. In addition, studies of the drug delivery systems based on the presented materials will be performed.

## 5. Conclusions

According to our results, all obtained materials can be safely used in biomedical applications, especially in biosensors due to low toxicity and good interferences with blood. Especially, a sample marked as SWCNH-ox-1.5, which is the purest studied material, has the least amorphous carbon residues (see Figure 4) and the largest internal specific surface area and pore volume [4]. Even in very low concentrations, good results of hemolysis, the possibility to maintain biocompatibility, and the best electrical properties proven in a previous paper [4] lead to the conclusion that this material will be the best for potential future biosensing applications, and this will be the subject of our forthcoming study.

## Figures and Tables

**Table 1:** Atomic concentration of C and O obtained from XPS measurements.

Atomic Concentration	SWCNH	SWCNH-ox-0.5	SWCNH-ox-1	SWCNH-ox-1.5	SWCNH-ox-3
C	97.79	92.4	90.0	90.3	88.4
O	2.21	7.6	10.0	9.7	11.6

## Data Availability

The data presented in this study are available on request from the corresponding author.
